# Strained Germanium Quantum Well PMOSFETs on SOI with Mobility Enhancement by External Uniaxial Stress

**DOI:** 10.1186/s11671-017-1913-3

**Published:** 2017-02-16

**Authors:** Yan Liu, Jiebin Niu, Hongjuan Wang, Genquan Han, Chunfu Zhang, Qian Feng, Jincheng Zhang, Yue Hao

**Affiliations:** 1 State Key Discipline Laboratory of Wide Band Gap Semiconductor Technology, Xidian University, Xi’an, 710071 China; 2 Laboratory of Nano-Fabrication and Novel Devices Integrated Technology, Institute of Microelectronics, Chinese Academy of Sciences, Beijing, 100029 China

**Keywords:** Germanium, MOSFET, Mobility, Quantum well

## Abstract

Well-behaved Ge quantum well (QW) p-channel metal-oxide-semiconductor field-effect transistors (pMOSFETs) were fabricated on silicon-on-insulator (SOI) substrate. By optimizing the growth conditions, ultrathin fully strained Ge film was directly epitaxially grown on SOI at about 450 °C using ultra-high vacuum chemical vapor deposition. In situ Si_2_H_6_ passivation of Ge was utilized to form a high-quality SiO_2_/Si interfacial layer between the high-κ dielectric and channels. Strained Ge QW pMOSFETs achieve the significantly improved effective hole mobility *μ*_eff_ as compared with the relaxed Si and Ge control devices. At an inversion charge density of *Q*_inv_ of 2 × 10^12^ cm^−2^, Ge QW pMOSFETs on SOI exhibit a 104% *μ*_eff_ enhancement over relaxed Ge control transistors. It is also demonstrated that *μ*_eff_ of Ge pMOSFETs on SOI can be further boosted by applying an external uniaxial compressive strain.

## Background

Germanium (Ge) has been attracting tremendous research interests for future pMOSFET applications due to it possesses the higher hole mobility over Si. Theoretical and experimental results proved that in order for Ge channel transistors to have significantly improved mobility and driving current over their Si and SiGe channel competitors, compressive strain is essential [1–3]. A great deal of efforts were devoted to demonstrating biaxially strained Ge-based ultrathin quantum well (QW) pMOSFETs [2, 4, 5], which have exhibited the advantages of confining hole in the undoped quantum well, eliminating dopant impurity scattering, and accommodating very high strain in channel. Nonetheless, the development of defect-free SiGe buffer with smooth surface on Si raised a major challenge for Ge devices. It was reported that, by optimizing the growth condition and controlling the film thickness precisely, fully strained Ge channel could be pseudomorphically grown directly on Si and silicon-on-insulator (SOI) [6–8]. With the low thermal budget device fabrication process, the strain in channel region was maintained, which substantially boosted the transistor performance. Studies showed that the uniaxial compressive strain is also promising for improving the mobility of Ge pMOSFETs [9–11]. However, there is still lack of the study on the combination effects between uniaxial and biaxial strain on Ge pMOSFETs.

In this paper, ultrathin strained Ge QW pMOSFETs on SOI are realized and characterized. Devices achieve the superior hole mobility to the relaxed Si and Ge control transistors. Electrical performance of Ge QW transistors is further improved by applying the external uniaxial compressive strain being parallel to channel direction.

## Methods

### Material Growth

**Fig. 1:**
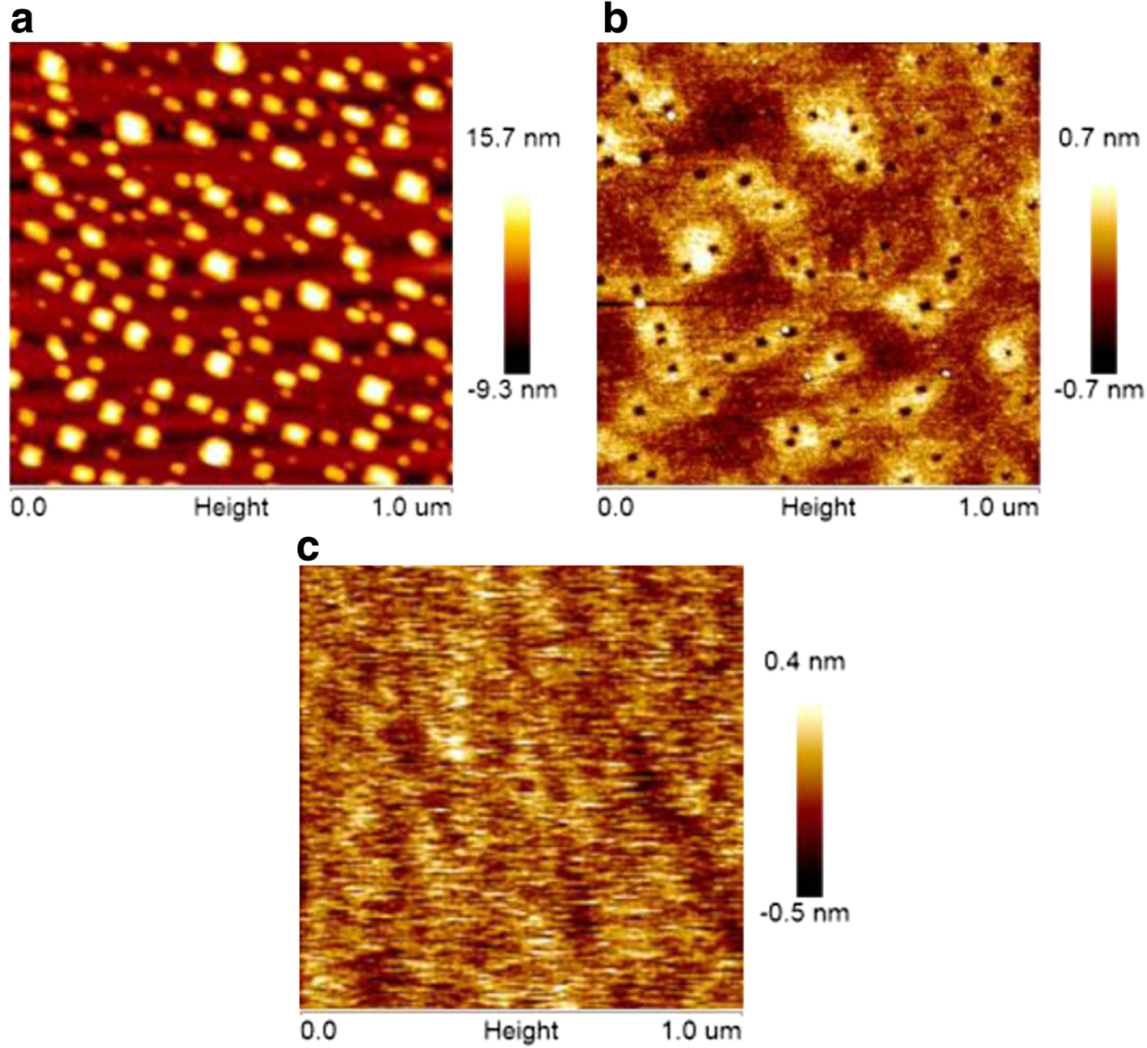
AFM images of epitaxially grown Ge on ultrathin SOI at **a** 500, **b** 400, and **c** 450 °C

Ge channel was epitaxially grown on lightly doped p-type SOI wafer using ultra-high vacuum chemical vapor deposition tool. Before loading into the growth chamber, the top Si layer was thinned down to about 7 nm using dry oxidation followed by the dilute HF etching. It is well known that, as Ge is epitaxially grown on Si, Ge islands tend to form in Stranski-Krastanow mode due to the 4.2% lattice mismatch between Ge and Si. The formation of Ge islands could be prevented by decreasing the growth temperature; nevertheless, the misfit dislocations are preferred to appear in Ge layer to release the strain energy. To achieve the continuous and defect-free epitaxial Ge film, we carried out the growth of Ge (the growth of Ge) on ultrathin SOI with different substrate temperatures. During the growth, the flow rate of GeH_4_ in a H_2_ carrier gas is 20 sccm, the pressure in growth chamber is about 10^−4^ Pa, and the growth duration is 5 min. Figure 1 shows the atomic force microscope (AFM) images of Ge film on SOI samples grown at various temperatures. The formation of large numbers of Ge islands was observed on the sample grown at 500 °C. With the reduction of growth temperature to 400 °C, it was found that many nanopits were yielded in Ge layer with the self-assembled growth, which partially released the strain energy. A flat surface was achieved for the Ge layer grown at 450 °C without the observation of any nanostructure. Experiments demonstrated that, as 3~5 nm Ge channel was epitaxially grown on SOI, the transition from 2 to 3D growth mode can be effectively suppressed with the growth temperature ranging from 420 to 450 °C.

### Device Fabrication

**Fig. 2:**
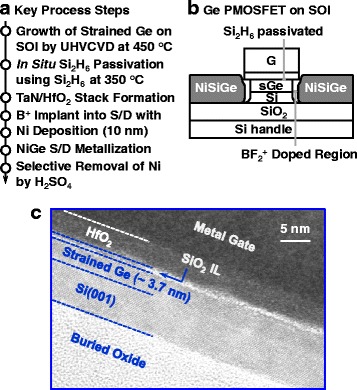
**a** Process sequence showing the key steps employed to fabricate the ultrathin fully strained Ge QW pMOSFET on SOI. **b** Cross-sectional view of a strained Ge pMOSFET with Si_2_H_6_ passivated interface. **c** HRTEM images of the gate stack on strained Ge channel on SOI. The thickness of Ge layer is 3.7 nm

The key process steps for fabricating ultrathin fully strained Ge QW pMOSFETs are shown in Fig. 2a. After the epitaxial growth of strained Ge layer, Ge channel was treated using in situ Si_2_H_6_ passivation at 350 °C for 15 min. Gate stack comprising hafnium dioxide (HfO_2_) by atomic layer deposition and metal gate by physical vapor deposition was formed. After the gate patterning and etching, BF_2_^+^ was implanted into drain and source regions at an energy of 10 keV and a dose of 1 × 10^15^ cm^−2^. Self-aligned metallic source/drain (S/D) was formed by depositing 10 nm nickel followed by a thermal anneal (RTA) at ~450 °C for 30 s. During the Ni germanosilicide, the p-type dopants in S/D regions were partially activated by the dopant segregation. The residual Ni was removed by the concentrated H_2_SO_4_ cleaning. Using the similar process, Si and Ge control pMOSFETs on bulk substrates were also fabricated.

Figure 2b shows the cross-sectional schematic of a fabricated Ge pMOSFET. Figure 2c depicts the high-resolution transmission electron microscope (HRTEM) image of metal gate stack on strained Ge channel on SOI. The thicknesses of defect-free Ge channel and HfO_2_ dielectric layer are 3.7 and 4.0 nm, respectively. Excellent interface quality and a uniform SiO_2_/Si interfacial layer (IL) are observed.

### Flexure-Based Bending Setup

**Fig. 3:**
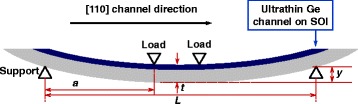
Schematic of the wafer bender for introducing uniaxial compressive strain to the devices along the channel direction

Uniaxial compressive strain was introduced into the Ge QW pMOSFETs on SOI through a mechanical flexure-based wafer bending apparatus. The apparatus is able to perform four-point bending upon chips positioned between loads and support, as shown in Fig. 3. The uniaxial strain was always applied parallel to the channel direction for the devices. The surface stress *σ* along the channel direction is calculated by *σ* = *E∙y∙t*/[2*a*∙(*L*/2 − 2*a*/3)], where *E* is the Young’s Modulus, *y* is the sample vertical displacement, *t* is the total thickness of the sample, *L* is the distance between the two supports, and *a* is the distance between the support and load [12]. The setup was calibrated with a load cell under the mounting platform and a strain gauge bonding on the sample.

Before measurement, the handle Si of SOI wafer was thinned down to about 300 μm by back side polishing to make sure it can accommodate the large strain. The values of Young’s modulus for Ge along [110] direction is 138 GPa [13].

## Results and Discussion

**Fig. 4:**
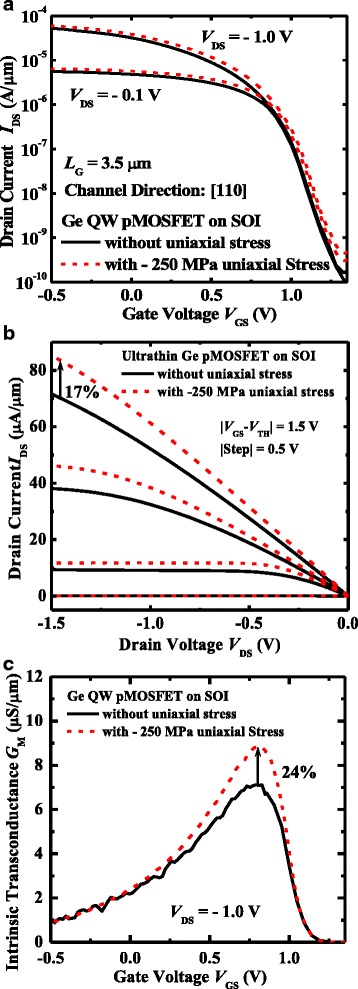
**a** Transfer and **b** output characteristics of a typical Ge QW pMOSFET on SOI with and without a −250 MPa uniaxial stress, the latter shows the drive current enhancement. **c** Devices under −250 MPa external uniaxial strain is observed to enhance the peak intrinsic transconductance by 24% over the Ge QW transistor without uniaxial strain

Figure 4a depicts the transfer characteristics of a typical Ge QW pMOSFET on SOI with and without external uniaxial compressive stress. The stress is along [110] and parallel to the channel direction. The gate length *L*_G_ of the device is 3.5 μm. Device has a subthreshold swing (SS) of ~90 mV/decade. The uniaxial stress has the negligible impact on the SS and the leakage floor characteristics of the Ge transistor. Here, *V*_TH_ is defined as the *V*_GS_ at a constant drive current of 10^−7^ A/μm and a *V*_DS_ of−0.1 V. *V*_TH_ is shown to be affected by the applied uniaxial compressive strain. As uniaxial stress is applied, devices exhibit a right shift of *V*_TH_. *I*_DS_−*V*_DS_ curves at different *V*_GS_–*V*_TH_ for the devices are illustrated in Fig. 4b, which demonstrate the *I*_DS_ enhancement in devices under the uniaxial compressive stress. A −250 MPa uniaxial stress provides a 17% Ge *I*_DS_ enhancement in ultrathin Ge pMOSFET on SOI at *V*_DD_ of 1.5 V. Figure 4c shows the linear intrinsic transconductance *G*_M_ of the same pair of transistors in Fig. 4a. At *V*_DS_ of −0.1 V, a 24% peak *G*_M_ is achieved in devices under −250 MPa external uniaxial strain compared to the Ge QW transistor without uniaxial strain.

**Fig. 5:**
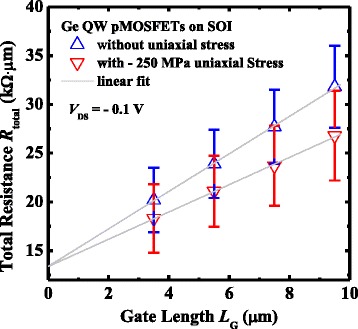
*R*_total_ as a function of *L*_G_ for the Ge QW transistors with and without external uniaxial compressive strain at *V*_GS_−*V*_TH_ of −1.5 V and *V*_DS_ of −0.1 V. The uniaxially strained pMOSFETs exhibit a smaller Δ*R*_total_/Δ*L*_G_ slope, indicating higher channel *μ*_eff_ compared to the devices without uniaxial compressive strain

To evaluate the intrinsic channel piezoresistance characteristics for the Ge QW pMOSFETs, large amount of devices with and without external uniaxial compressive strain were measured with *L*_G_ ranging from 3.5 to 9.5 μm. The total resistance *R*_total_ as a function of *L*_G_ extracted at a gate overdrive of −1.5 V and *V*_DS_ of −0.1 V are plotted in Fig. 5. The intercept of the fitted lines with the *y*-axis yields the value of source/drain resistance *R*_SD_. *R*_SD_ of Ge QW pMOSFETs on SOI is about 14 kΩ μm, and it is observed that the external uniaxial stress has little impact on *R*_SD_. The slope of Δ*R*_total_/Δ*L*_G_ represents the channel resistance *R*_ch_, which is related to the effective hole mobility *μ*_eff_ and inversion charge density. The uniaxially strained pMOSFETs achieve a 24% reduction in Δ*R*_total_/Δ*L*_G_ slope i.e. *R*_ch_, compared to the devices without uniaxial compressive strain. Experiment demonstrates that the impact of external uniaxial strain on inversion capacitance of Ge QW pMOSFETs is negligibly small. Therefore, the decreasing of *R*_ch_ is attributed to the improvement in *μ*_eff_ for the transistors with the external uniaxial compressive stress.

**Fig. 6:**
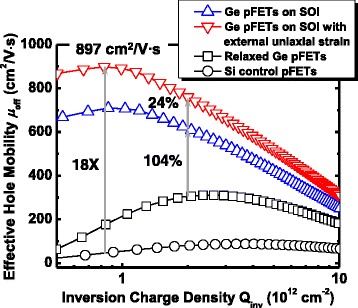
*μ*_eff_ versus *Q*_inv_ for Ge QW pMOSFETs on SOI with and without a −250 MPa uniaxial stress. Ge pMOSFETs on SOI demonstrate a 104% *μ*_eff_ improvement over relaxed Ge control device at the *Q*_inv_ of 2 × 10^12^ cm^−2^. The *μ*_eff_ of the devices under −0.18% uniaxial strain along [110] channel direction is further improved by 24%. Ge QW pMOSFETs on SOI with uniaxial compressive strain obtain a peak *μ*_eff_ of 897 cm^2^/V s. Eighteen times higher *μ*_eff_ over Si control devices is achieved in uniaxially strained Ge QW pMOSFETs at low *Q*_inv_

*μ*_eff_ is a crucial factor, which affects the drive current and *G*_M_ of the devices. We extracted the *μ*_eff_ using the Δ*R*_total_/Δ*L*_G_ method. *μ*_eff_ was calculated by *μ*_eff_ = 1/[*WQ*_inv_(Δ*R*_total_/Δ*L*_G_)], where *W* is the channel width, *Q*_inv_ is the inversion charge density in Ge QW channel, and Δ*R*_tot_/Δ*L*_G_ is the slope of the *R*_total_ as a function of *L*_G_ plots as shown in Fig. 5. *Q*_inv_ in the channel was calculated by integrating the measured *C*_inv_ versus *V*_GS_ curves. Figure 6 illustrates the *μ*_eff_ as function of *Q*_inv_ characteristics of the Ge QW pMOSFETs on SOI with and without a −250 MPa uniaxial stress. Comparison of *μ*_eff_ for the Ge QW pMOSFETs with Si and Ge control on bulk wafers is also presented. Ge QW pMOSFETs under uniaxial compressive strain achieve a peak *μ*_eff_ of 897 cm^2^V^−1^s^−1^. At the low *Q*_inv_, Ge QW pMOSFETs under uniaxial compressive strain demonstrate an 18 times higher *μ*_eff_ as compared with Si control devices. At the *Q*_inv_ of 2 × 10^12^ cm^−2^, Ge QW pMOSFETs without external uniaxial strain demonstrate a 104% *μ*_eff_ enhancement of over the relaxed Ge control device. This is attributed to the fact that the biaxial compressive strain in ultrathin Ge channel leads to the splitting of valence bands, and top valence band populated by carrier has a reduced effective mass than relaxed Ge [14, 15]. It is noted that, under the external uniaxial compressive strain, the Ge QW devices demonstrate a 24% *μ*_eff_ enhancement in comparison with the transistors without external strain. It is speculated that the external uniaxial compressive strain further decreases the effective hole mobility, leading to the additional mobility enhancement, along transport direction in strained Ge channel.

## Conclusions

High-mobility strained Ge QW pMOSFETs with high channel crystallinity on SOI platform are realized. Devices exhibit good transfer and output characteristics and a significantly improved *μ*_eff_ over Si and Ge control pMOSFETs. Ge QW pMOSFETs on SOI obtain a 104% improvement in *μ*_eff_ in comparison with the relaxed Ge control transistors at a fixed *Q*_inv_ of 4 × 10^12^ cm^−2^. Ge QW pMOSFETs on SOI under an external uniaxial compressive stain achieve a further *μ*_eff_ enhancement, contributing to the reduced *R*_ch_ and the improved drive current over the transistors without external uniaxial compressive stain.
